# Bigeminy with Prolonged QT Interval as an Ominous Sign for Impending Torsades de Pointes: A Case Report

**DOI:** 10.5811/cpcem.19484

**Published:** 2024-11-18

**Authors:** Thuc Vu, Jake Valentine

**Affiliations:** * Kansas City University College of Osteopathic Medicine, Kansas City, Missouri; † University of Houston Tilman J. Fertitta Family College of Medicine, Houston, Texas; ‡ HCA Houston Healthcare Kingwood, Kingwood, Texas

**Keywords:** torsades de pointes, long QT, case report, bigeminy

## Abstract

**Introduction:**

Ventricular ectopic beats and corrected QT interval (QTc) prolongation are both relatively common entities that are typically benign. It is difficult to predict subsequent dysrhythmias from either electrocardiogram (ECG) feature. The combination of both features may better predict the risk of torsades de pointes. We highlight a case of torsades preceded by a bizarre bigeminal rhythm with QTc prolongation likely caused by memantine use and hypokalemia.

**Case Report:**

An 84-year-old female presented to the emergency department with a fall. A syncope workup revealed an ECG demonstrating bigeminy with a prolonged QTc interval. Several minutes after obtaining the ECG, the patient went into torsades. She had multiple subsequent cardiac arrests during the rest of her hospital stay. This case report details the importance of recognizing ventricular bigeminy in the context of QTc prolongation as a harbinger of torsades.

**Conclusion:**

While premature ventricular contractions including bigeminy may be a benign finding, when accompanied by prolonged QTc intervals, they warrant immediate investigation and treatment of potential underlying pathology to prevent torsades and subsequent cardiac arrest.

## INTRODUCTION

Ventricular ectopic beats and corrected QT interval (QTc) prolongation are both relatively common entities that are typically benign. It is difficult to predict subsequent dysrhythmias from either electrocardiogram (ECG) feature. The combination of both features may better predict risk of torsades de pointes.^1^ We highlight a case of torsades preceded by a bizarre bigeminal rhythm with QTc prolongation likely caused by memantine use and hypokalemia. The report includes teaching points on the pathophysiology of torsades and the clinical correlation to typical ECG changes predicting development of torsades beyond the traditional prolonged QTc and “R on T” phenomenon frequently cited in emergency medicine textbooks.

## CASE REPORT

**Image 1:**
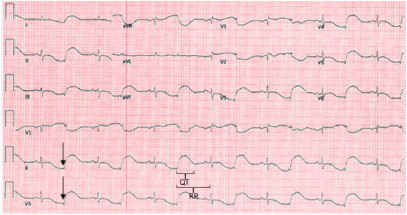
Electrocardiogram revealing sinus rhythm with bizarre-appearing bigeminy. The subtle low-voltage QRS complexes best seen in leads II and V5 (arrows) represent premature ectopic beats. Heart rate is 85 beats per minute, and the computer reported a QTc of 602 milliseconds. It is difficult to delineate the end of the T-wave in the sinus beat, but the QT interval for the ectopic beat is larger than half the RR interval (brackets), supporting the diagnosis of prolonged QTc.

An 84-year-old female presented to the emergency department (ED) due to a laceration to her forehead. She had a history of dementia, chronic kidney disease, and hypertension, for which she was prescribed memantine and amlodipine. She had been in her usual state of health the night prior but walked downstairs the morning of her ED presentation due to blood trickling down her face. She could not recall whether she had fallen or provide any meaningful details surrounding the event. She denied any other acute medical complaint. The patient’s family had not noticed any outward evidence of illness prior to discovering the wound and reported that the patient was at her baseline mental state. The patient was alert, with a non-focal neurologic exam, and had an isolated two-centimeter laceration to the middle of her forehead without active bleeding. She had a regular rate and rhythm but bizarre-appearing morphology on her telemetry monitoring. The emergency physician relayed the plan for trauma and syncope workups to include computed tomography (CT) of the head and cervical spine, tetanus vaccination, wound irrigation and preparation for closure, along with basic labs and an ECG, the result of which is below (Image 1).

After reviewing the ECG, the physician ordered two grams of magnesium based on the “bizarre repolarization pattern with QTc of 602 milliseconds.” Intravenous access was obtained, magnesium was started, and the patient was brought expeditiously to CT at which point she went into cardiac arrest, with the bedside nurse reporting ventricular fibrillation as the initial rhythm. The patient achieved return of spontaneous circulation (ROSC) after a single defibrillation and two-minute round of compressions per Advanced Life Support (ALS) protocol. The physician also requested the rest of the magnesium to be delivered at a bolus rate. The patient maintained her baseline neurologic exam after the cardiac arrest. Her post-arrest ECG revealed sinus rhythm without ectopy at a rate of 107 beats per minute with a partial right bundle-branch block, a QTc of 451 that was difficult to confirm by visual inspection due to diffuse T-wave flattening, and no specific evidence of ischemia.

**Image 2:**
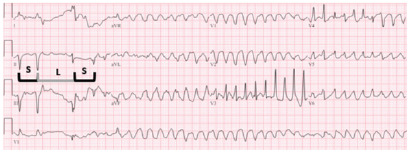
12-lead electrocardiogram (ECG) captured during development of torsades. The ECG displays the classic “short-long-short” sequence prior to development of the dysrhythmia, with “S” representing the short RR interval and “L” representing the long RR interval.

Notable lab results included a potassium of 3.1 millimoles per liter (mmol/L) (reference range 3.5–5.1 mmol/L), magnesium of 1.9 milligrams per deciliter (mg/dL) (1.8–2.4 mg/dL), and minimally elevated troponin of 171 picograms per milliliter (pg/mL) (0–53 pg/mL). Amiodarone had initially been held in order not to propagate QTc prolongation but was initiated at time of lab result along with potassium repletion, given the unexpectedly mild electrolyte derangements and the computer-determined correction of the QTc interval. Several hours later, while awaiting an intensive care unit (ICU) bed, the patient suffered another cardiac arrest, this time captured on telemetry monitoring, which showed torsades (Image 2).

**CPC-EM Capsule**

What do we already know about this clinical entity?

*A prolonged QT interval is described as a classic risk factor for torsades de pointes*.

What makes this presentation of disease reportable?

*This case highlights useful predictors of torsades de pointes that align with the proposed pathophysiology of this rare disease process*.

What is the major learning point?

*The combination of frequent ectopy and prolonged QT interval multiplies the risk of torsades de pointes*.

How might this improve emergency medicine practice?

*Sharing this case will raise awareness of the risk factors and emergency management of torsades de pointes*.

The patient again achieved ROSC with return of baseline neurological status after a single defibrillation and round of compressions. A 300 mg bolus of amiodarone was given at this time at the direction of the ICU physician, and she was promptly transferred to the ICU. Her hospital stay was complicated by at least four more episodes of documented torsades. Her repeat troponin several hours later remained adynamic at 180 pg/mL. She suffered progression of her chronic kidney disease to oliguric renal failure, requested a “do not resuscitate” order, and was discharged to hospice care several days later.

## DISCUSSION

**Image 3:**
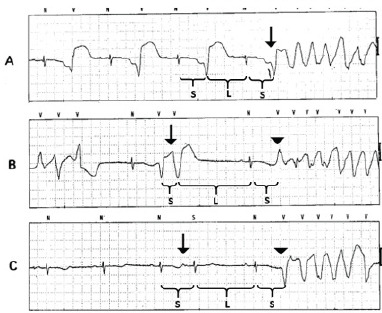
(Adapted, with permission, from Locati et al, 1995^2^): Examples of “short-long-short” RR intervals preceding torsades. Brackets denote the RR intervals of interest, with “S” signifying a short interval and “L” signifying a long interval. In example A, there are alternating short and long intervals owing to bigeminy. The premature ventricular contraction (PVC) initiating torsades strikes imperceptibly earlier (arrow), fulfilling the “short-long-short” pattern. In example B, the second of a two-run beat of PVCs (arrow) creates the first short interval. A compensatory pause until the next sinus beat creates the long interval, followed by the initiating PVC (arrowhead) completing another “short-long-short” sequence. Example C is started by a premature atrial contraction (arrow), creating the first short interval. Again, a compensatory pause follows, with another sinus beat and subsequent PVC (arrowhead) initiating torsades.

This case underscores the critical importance of recognizing the association of bigeminy with a markedly prolonged QTc interval as a sign of impending torsades. Ventricular ectopy is perhaps the best predictive marker for torsades in the setting of a prolonged QT interval.^1^ In a retrospective review of Holter-monitor-captured torsades, 103 of 105 episodes were caused by a “short-long-short” RR interval, which is elaborated upon below (Image 3).^2^

There appear to be at least two theories as to why the appearance of bigeminy with a long QT interval is an ominous sign for torsades. The “early after-depolarizations” explanation attributes bigeminy to a profound repolarization disturbance in which electrical instability across the repolarizing cardiac membrane triggers another depolarization, creating the bigeminal beat.^3^

An alternate theory directly implicates bigeminy as the cause of torsades.^3^ In this theory, bigeminy is caused by a competing parasystolic focus. The independent depolarizations create alternating RR intervals, which predispose the patient to the “R on T” phenomenon. This phenomenon is the pathophysiologic underpinning of the “short-long-short” sequences detailed above (Image 3). In simpler terms, the alternating “short-long” sequence entailed by bigeminy creates opportunity for a “short-long-short” sequence to trigger torsades. A prolonged QTc creates an opportunity during the “long” sequence for the early arrival of the subsequent depolarization to fall during the critical period of repolarization. This might explain why only the combination, and neither bigeminy nor QTc prolongation alone, markedly increases the risk of torsades.

Our patient’s QTc prolongation may have been medication-induced and exacerbated by hypokalemia. Memantine has been implicated in QTc prolongation in several case reports.^4^,^5^ In the case of bigeminy, the QTc interval associated with the longer RR interval ought to be measured, as this is the interval that will provoke torsades if a subsequent depolarization occurs during the “long” cycle repolarization. When in doubt, use the longest QTc measurement from the lead with the most clearly identified T-wave. Physicians must be careful of the case with indiscernible T-waves due to diffuse flattening or other morphologic abnormalities as QT measurements will be unreliable. In cases in which the QT interval appears longer than half the RR interval, a normal computer-derived QTc measurement should be checked manually using Bazett’s formula.^6^

Critical actions for the presenting ECG would include magnesium, potassium repletion, and avoidance of QTc-prolonging agents. Torsades management includes standard ALS with the exception of amiodarone, and the additional level 1-B recommendation for overdrive pacing or isoproterenol administration in cases refractory to magnesium.^7^ In hindsight, we would have delayed CT in this patient without a focal neurologic deficit until the electrolytes had resulted and magnesium and potassium had finished infusing. We would have withheld amiodarone after ROSC, recognizing that the QTc on the post-ROSC ECG was indeterminate due to diffuse T-wave flattening. We would have advocated harder against the additional amiodarone ordered by the ICU team and recommended instead to consider overdrive pacing or isoproterenol administration. We hope that the lessons learned from this case can help our readers identify and manage this uncommon ECG pattern in the future.

## CONCLUSION

While premature ventricular contractions including bigeminy may be a benign finding, when accompanied by prolonged QTc intervals they warrant immediate investigation and treatment of potential underlying pathology to prevent torsades and subsequent cardiac arrest.
